# Administration of PDE4 Inhibitors Suppressed the Pannus-Like Inflammation by Inhibition of Cytokine Production by Macrophages and Synovial Fibroblast Proliferation

**DOI:** 10.1155/2007/58901

**Published:** 2007-09-27

**Authors:** Katsuya Kobayashi, Toshio Suda, Haruhiko Manabe, Ichiro Miki

**Affiliations:** Pharmacological Research Laboratories, Pharmaceutical Research Center, Kyowa Hakko Kogyo Co., Ltd., 1188 Shimotogari, Nagaizumi-Cho, Sunto-Gun, Shizuoka-Ken 411-8731, Japan

## Abstract

A marked proliferation of synovial fibroblasts in joints leads to pannus formation in rheumatoid arthritis (RA). Various kinds of cytokines are produced in the pannus. The purpose of this study is to elucidate the effects of phosphodiesterase 4 (PDE4) inhibitors in a new animal model for the evaluation of pannus formation and cytokine production in the pannus. Mice sensitized with methylated bovine serum albumin (mBSA) were challenged by subcutaneous implantation of a membrane filter soaked in mBSA solution in the back of the mice. Drugs were orally administered for 10 days. The granuloma formed around the filter was collected on day 11. It was chopped into pieces and cultured in vitro for 24 hr. The cytokines were measured in the supernatants. The type of cytokines produced in the granuloma was quite similar to those produced in pannus in RA. Both PDE4 inhibitors, KF66490 and SB207499, suppressed the production of IL-1β, TNF-α, and IL-12, and the increase in myeloperoxidase activity, a marker enzyme for neutrophils and hydroxyproline content. Compared to leflunomide, PDE4 inhibitors more strongly suppressed IL-12 production and the increase in myeloperoxidase activity. PDE4 inhibitors also inhibited lipopolysaccharide-induced TNF-α and IL-12 production from thioglycolate-induced murine peritoneal macrophages and the proliferation of rat synovial fibroblasts. These results indicate this model makes it easy to evaluate the effect of drugs on various cytokine productions in a granuloma without any purification step and may be a relevant model for evaluating novel antirheumatic drugs on pannus formation in RA. PDE4 inhibitors could have therapeutic effects on pannus formation in RA by inhibition of cytokine production by macrophages and synovial fibroblast proliferation.

## 1. INTRODUCTION

Rheumatoid arthritis (RA) is a systemic autoimmune disease characterized by the progressive chronic inflammation of multiple joints. The incidence of RA is close to 1% worldwide, but its etiology is not yet known. The search for therapeutic agents for RA is being conducted mainly using collagen-induced arthritis and adjuvant-induced arthritis [1, 2]. In the development of arthritis, pannus formation is the critical step in the destruction of cartilage and bones. Pannus is a proliferative granulomatous tissue, mainly composed of macrophages and fibroblast-like synoviocytes. Pannus contains numerous cytokines and proteases inside. Although collagen-induced arthritis and adjuvant-induced arthritis models are very useful for screening candidates for novel antirheumatic drugs, it is not easy to elucidate the effects of candidates on the pannus formation. Also, quantitative analysis of cytokine levels is required to elucidate the role of cytokines in the pathogenesis of pannus formation. However, many steps are needed to measure cytokines in the pannus in arthritis models. For example, the ankle joints have to be removed, frozen in liquid nitrogen, crushed, homogenated, and finally cytokines are assayed by enzyme-linked immunosorbent assay (ELISA) in the supernatants [3]. Therefore, an inflammatory model is needed to simply evaluate the effects of drugs on pannus formation and cytokine production in pannus.

There is only a few animal models available for the evaluation of antiarthritic drugs on pannus formation. One of the granuloma models, a cotton pellet-induced granuloma model, is widely used as a model for pannus formation [4]. The problem with this model is, however, that the granuloma is formed by a foreign-body-dependent granuloma reaction. The mechanism of pannus formation has not been fully proven yet, but T cells seem to be involved in pannus formation [5]. Another granuloma model, delayed-type hypersensitivity (DTH) granuloma, has been reported to be antigen-specific and T cell-mediated [6–8]. Histological features of the lesions from DTH granuloma revealed the evolution of DTH into a chronic glaucomatous reaction, as shown by the following changes: fibrin deposition, vasculitis, mononuclear cell infiltration/proliferation, and angiogenesis [6]. They reported that DTH granuloma represented a relevant model for probing pathogenic mechanisms and potential therapeutics for RA. In this study, we modified the DTH granuloma model so that we could easily measure cytokine production from the granulomatous tissue. The granuloma was induced by a subcutaneous implantation of a thin membrane filter soaked in methylated bovine serum albumin (mBSA) solution into the back of the mice previously sensitized with mBSA. For cytokine determination, the granuloma was collected from the back of the mice on the indicated days and incubated in culture medium for 24 hours in vitro, and the cytokines were measured in the supernatants.

Phosphodiesterase 4 (PDE4) inhibitors are key enzymes that degrade cAMP and play an important role in inflammatory and immune reactions [9]. The prevention of cAMP degradation by PDE4 inhibitors elevates the level of cAMP in the cells followed by suppression of inflammation and immune responses. Rolipram is a well-known PDE4 inhibitor that reduces the inflammatory responses in several rodent models, including carrageenan-induced edema [10], adjuvant arthritis [10, 11], antigen-induced airway inflammation [12, 13], lipopolysaccharide- (LPS-) induced lung inflammation [14] and collagen-induced arthritis [15]. Inhibitions of leukotriene B_4_ (LTB_4_), interferon-*γ* (IFN-*γ*), tumor necrosis factor-*α* (TNF-*α*), interleukin-4 (IL-4), and IL-5 are all potential mechanisms by which the anti-inflammatory effects of rolipram are mediated [16]. These results suggest that PDE4 inhibitors may be therapeutic agents for various diseases such as asthma, chronic obstructive pulmonary disease, and rheumatoid arthritis. In this study, we evaluated PDE4 inhibitors, KF66490 and SB207499 [17], in the mouse pannus model and compared the effects of the PDE4 inhibitors with those of typical antirheumatic drugs leflunomide, prednisolone, and methotrexate.

## 2. MATERIAL AND METHODS

### 2.1. Animals

Male, 6-week-old BALB/c mice were purchased from Charles River Japan (Kanagawa, Japan) and maintained at a temperature of $22\pm 3^{{}^{\circ}}\text{C}$ and a humidity of 50±20%. Food and water were provided ad libitum. The study protocol for the animal experiment was approved by the Animal Care Committee of Kyowa Hakko Kogyo Co., Ltd.

### 2.2. Reagents

Leflunomide, KF66490 and SB207499 were synthesized in our institute. Methotrexate (MTX) was purchased from Lederle Japan, Ltd. (Tokyo, Japan). Prednisolone, diclofenac sodium and methylated bovine serum albumin (mBSA) were purchased from Sigma-Aldrich Japan (Tokyo, Japan). Sources of other chemicals were as follows: Freund's complete adjuvant (Difco, Detroit, Mich, USA); myeloperoxidase (Sigma-Aldrich Japan), hexadecyltrimethylammonium bromide (Sigma-Aldrich Japan); ELISA kits for each mouse cytokine: IL-6 and IL-1*β* (BioSource International, Camarillo, Calif, USA), TNF-*α* (R*&*D Systems, Minneapolis, MN, USA), IFN-*γ* (PIERCE, Rockford, Ill, USA), IL-4 (BD Bioscience, San Diego, Calif, USA), IL-10 (BD Bioscience), IL-12 (Genzyme, Cambridge, Mass, USA); RPMI 1640 tissue culture medium (Sigma-Aldrich); fetal bovine serum (Intergen, Purchase, NY, USA); penicillin-streptomycin (Life Technologies, Rockville, Md, USA); and 2-mercaptoethanol (Wako Pure Chemical).

### 2.3. Induction of granuloma

Granuloma was induced according to Dunn et al. [6] with a slight modification. Methylated BSA (mBSA: 4 mg/mL in saline) was emulsified in an equal volume of Freund's complete adjuvant. The emulsion (0.1 mL containing 200 *μ*g mBSA) was injected intradermally on the back of the mice. Seven days later, the same treatment was repeated. Two weeks after the last sensitization, a membrane filter (13 mm diameter, 0.05 mm thickness, Millipore Japan, Tokyo, Japan) which had been soaked in mBSA solution (4 mg/ml in saline) for 60 minutes was subcutaneously implanted in the dorsum of the mice (day 0). The wound was closed with 9 mm autoclips (Clay Adams, Parsippany, NJ).

### 2.4. Measurement of cytokines and anti-mBSA antibodies in the culture supernatants

Mice were killed by CO_2_ asphyxiation. The granuloma formed around the filter was collected from the back of the mice and chopped into pieces in 4 mL of 1640 tissue culture medium supplemented with 10% heat-inactivated fetal bovine serum (FBS), 50 unit/mL penicillin-50 *μ*g/mL streptomycin, and 50 *μ*mol/L 2-mercaptoethanol. The tissue was cultured for 24 hours at 37^*°*^C. Cytokines were measured by ELISA kits in the supernatants. Anti-mBSA specific IgG_1_ and IgM antibodies in the supernatants were measured by ELISA [18], and the results were expressed as the absorbance at 450 nm.

### 2.5. Measurement of myeloperoxidase activity and hydroxyproline content in the granuloma

Myeloperoxidase activity was measured using a specific substrate in the mixture of the culture medium and the granulomatous tissue. Briefly, the chopped tissues and the remaining supernatants (total 3.2 mL) were mixed with 0.16 mL of potassium phosphate buffer (0.5 mol/L, pH 6.0) and 0.16 mL hexadecyltrimethylammonium bromide solution (10%) and vigorously vortexed. Samples were incubated for 20 minutes at 4^*°*^C. After centrifugation (1800 g, 20 minutes), the enzyme activity in the supernatant was measured [19]. The remaining tissue and the supernatant were mixed with an equal amount (3.3 mL) of HCl (12 mol/L) and incubated for 15 hours at 110^*°*^C for the measurement of hydroxyproline content according to the procedure of Jakubzick [20]. Data were expressed as total enzyme activity or total hydroxyproline content in the granuloma.

### 2.6. Time course analysis of the murine pannus model

The granuloma was collected on days 1, 3, 6, 10, 15, 22, and 30 and cultured in vitro. A saline-soaked filter was implanted in the back of the mice as a negative control group (Saline group).

### 2.7. Evaluation of antirheumatic drugs and PDE4 inhibitors

All drugs were suspended in 0.5% methylcellulose in distilled water and orally administered once a day from day 0 to 10. Control group was treated with 0.5% methylcellulose (Vehicle group). The granuloma was collected on day 11.

### 2.8. Thioglycolate-elicited peritoneal macrophages

Male BALB/c mice (8–10 weeks old) were injected intraperitoneally with 1 ml of 3% (w/vol) thioglycolate broth (Wako Pure Chemical). Four days later, cells were harvested by peritoneal lavage with RPMI 1640 medium. The cells were washed twice, and $2.5\times 10^{4}$ peritoneal cells (for TNF-*α* production) or $2\times 10^{5}$ peritoneal cells (for IL-12 production) per 0.2 mL in the same medium containing 10% FBS were then added to each well of 96-well plates. The cells were allowed to adhere to well at 37^*°*^C in a humidified 5% CO_2_ atmosphere, and after 2 hours, nonadherent cells were removed by gentle washing. Adherent cells were used as thioglycolate-elicited peritoneal macrophages. Adherent cells were stimulated with 1 *μ*g/mL of LPS for 6.5 hours (for TNF-*α*) or 24 hours (for IL-12) at 37^*°*^C. After the incubation, the supernatant was collected. Cytokines were measured by ELISA kits in the supernatants.

### 2.9. Culture of rat synovial fibroblasts

Surface parts of synovial tissues were isolated from knee joints of female, 8-week-old Lewis rats (Charles River Japan), followed by washing with phosphate-buffered saline (PBS) and subsequent digestion with 0.2% collagenase in RPMI 1640 medium at 37^*°*^C for 2 hours. Synovial tissues were then treated with 0.2% collagenase and 0.25% trypsin at 37^*°*^C for 2 hours. Cells were collected in RPMI 1640 containing 10% FBS and antibiotics, and then centrifuged at 500 x*g* for 5 minutes. The pellets were suspended in RPMI 1640 containing 10% FBS. Cells were used for experiments after three to eight passages as synovial fibroblasts.

### 2.10. Proliferation of rat synovial fibroblasts

Synovial fibroblasts ($1\times 10^{5}$ cells) suspended in 200 *μ*L of RPMI 1640 medium supplemented with 1% FBS, 0.9% BSA and antibiotics were precultured for 24 hours at 37^*°*^C. After the preculture, the culture medium was changed to RPMI 1640 medium supplemented with 10% FBS and cells were cultured in the presence or absence of compounds for 48 hours at 37^*°*^C followed by 24 hours culture in the presence of 1.25 *μ*Ci/mL of [methyl-^3^H] thymidine. In the case of methotrexate and leflunomide, 1.25 *μ*Ci/mL of [6-^3^H] deoxyuridine was also used instead of [methyl-^3^H] thymidine [21, 22]. The incorporation of [^3^H] thymidine or [6-^3^H] deoxyuridine was counted by a liquid scintillation counter.

### 2.11. Statistical analyses

The Student's t test or Aspin-Welch test following an F-test was used for analysis of differences between the two groups. Multiple comparisons among treatment groups were performed by 1-way ANOVA followed by the Dunnett test or Kruskal-Wallis followed by the Steel test. P values less than .05 were considered to be statistically significant.

## 3. RESULTS

### 3.1. Time course analysis of the murine pannus model

**Figure 1:**
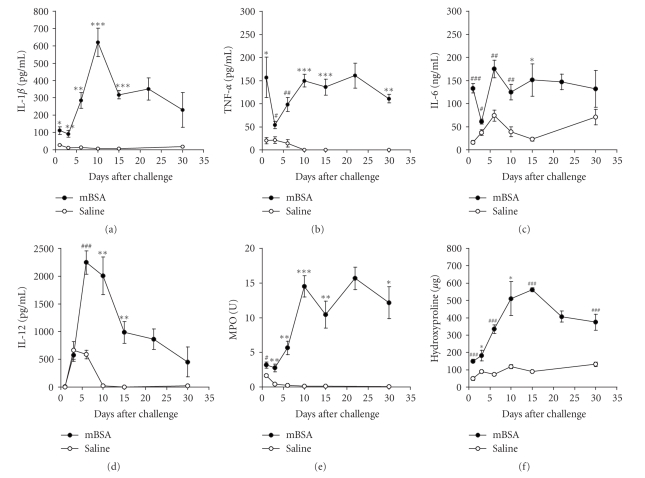
Time course of cytokine productions, myeloperoxidase (MPO) activity, and hydroxyproline content in the mBSA-induced granulomatous tissue. Mice sensitized with mBSA were challenged by subcutaneous implantation of a membrane filter soaked in mBSA solution in the back of the mice on day 0. Granuloma was collected on the indicated days and cultured in vitro for 24 hr. Cytokines were measured in the supernatants. MPO activity and hydroxyproline content were measured in a mixture of the culture medium and the granuloma. IL-1*β* (a), TNF-*α* (b), IL-6 (c), IL-12 (d), MPO activity (e), and hydroxyproline content (f). Results are shown as the mean ± SE of 4 to 6 mice. *#*: P<.05, *#* *#*: P<.01, *#* *#* *#*: P<.001 (The Student's t test), *P<.05, **P<.01, **P<.001 (Aspin-Welch test). Representative data from three independent experiments are shown.

The granulomas collected on the indicated days were chopped into pieces in culture medium. Cytokine concentrations and anti-mBSA specific IgG_1_ and IgM antibody titer in the supernatants were measured after 24-hour incubation without any manipulation to extract cytokines from the granulomatous tissues (Figure 1). The concentration of IL-1*β* peaked on day 10 and then gradually decreased in the mBSA-treated group (Figure 1(a)). TNF-*α* and IL-6 were both released on day 1, transiently decreased on day 3, and were constantly released until day 30 (Figures 1(b), 1(c)). IL-12 release peaked on day 6, while IFN-*γ* was slightly delayed and peaked on day 10 (Figure 1(d) and data not shown). IL-4 and IL-10 were significantly increased only on day 1 (data not shown). Anti-mBSA specific IgG_1_ antibody concentration was significantly increased from day 6 and sustained until day 22 (data not shown). Anti-mBSA specific IgM antibody concentration was significantly increased from day 1 and peaked on day 15 (data not shown). Myeloperoxidase (MPO) activity, a marker enzyme for neutrophil infiltration in the mixture of the culture medium and the granuloma, was constantly high in the mBSA-treated group (Figure 1(e)). Hydroxyproline content, a marker of fibrosis, increased with time and peaked on day 15 in the granuloma (Figure 1(f)).

### 3.2. Evaluation of antirheumatic drugs and PDE4 inhibitors

**Figure 2:**
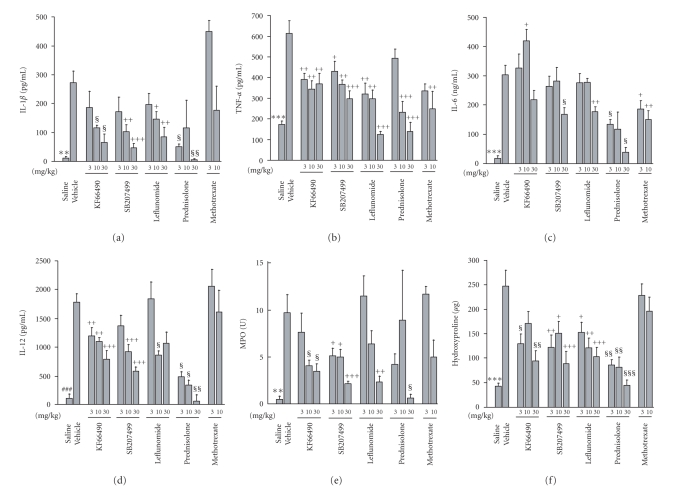
Effects of antirheumatic drugs and a PDE4 inhibitor on the mBSA-induced granuloma. Drugs were orally administered once a day from day 0 to 10. On day 11, the granuloma was collected and cultured for 24 h at 37^*°*^C. Cytokine concentrations in the supernatants and MPO activity and hydroxyproline content in the mixture of the culture medium and the granuloma were measured. PDE4 inhibitors, KF66490 (3, 10, 30 mg/kg) and SB207499 (3, 10, 30 mg/kg), leflunomide (3, 10, 30 mg/kg), prednisolone (3, 10, 30 mg/kg), and methotrexate (3, 10 mg/kg) were orally administered. IL-1*β* (a), TNF-*α* (b), IL-6 (c), IL-12 (d), MPO activity (e), and hydroxyproline content (f). Results are shown as the mean ± SE of 6 to 10 mice. *#* *#* *#*: P<.001 (The Student's t test), **P<.01, ***P<.001 (Aspin-Welch test), +: P<.05, ++: P<.01, +++: P<.001 (Dunnett test), *§*: P<.05, *§* *§*: P<.01, *§* *§* *§*: P<.001 (Steel test). Representative data from three independent experiments are shown.

Drugs were orally administered for 10 days from the day of the implantation of mBSA-soaked filter in the back of the mice. IL-1*β* production from the granuloma was suppressed by both PDE4 inhibitors, KF66490 and SB207499, leflunomide, and prednisolone, but not by methotrexate (Figure 2(a)). TNF-*α* production was suppressed by all drugs (Figure 2(b)). IL-6 production was suppressed by all drugs except KF66490 (Figure 2(c)). IL-12 production was suppressed by both PDE4 inhibitors and prednisolone and partially by leflunomide but not by methotrexate (Figure 2(d)). Increase in MPO activity was suppressed by all drugs except for methotrexate (Figure 2(e)). Increase in hydroxyproline content was suppressed by all drugs except methotrexate (Figure 2(f)). Summary of the data in this study are shown in Tables 1and2. PDE4 inhibitors showed a wide range of effects in this model. PDE4 inhibitors suppressed anti-mBSA specific IgG_1_ antibody production, but not anti-mBSA specific IgM antibody production. Leflunomide and methotrexate strongly suppressed both IgG_1_ and IgM antibody production. Nonsteroidal anti-inflammatory drugs (NSAIDS), diclofenac significantly increased the TNF-*α* and IL-12 production. None of the drugs affected the body weight increase except prednisolone, which significantly suppressed it both at 10 and 30 mg/kg (data not shown).

### 3.3. Cytokine production from thioglycolate-elicited peritoneal macrophages

To evaluate the effects of PDE4 inhibitors on proinflammatory cytokine production, LPS-stimulated TNF-*α* and IL-12 production from thioglycolate-elicited peritoneal macrophages were conducted in vitro. Both PDE4 inhibitors suppressed LPS-stimulated TNF-*α* and IL-12 production with IC_50_ values of 0.88 and 4.2 *μ*mol/L (KF66490), and 0.12 and 0.52 *μ*mol/L (SB207499), respectively (Table 3).

### 3.4. Proliferation of rat synovial fibroblasts

To evaluate the antiproliferative activity of PDE4 inhibitors and antirheumatic drugs on fibroblasts, the proliferation of rat synovial fibroblasts was conducted. KF66490 and SB207499 inhibited serum-stimulated proliferation of rat synovial fibroblasts with an IC_50_ values of 9.2 and 3.1 *μ*mol/L, respectively (Table 4). Leflunomide did not inhibit it even at 100 *μ*mol/L. Prednisolone and methotrexate inhibited it with IC_50_ values of 0.070 and 0.013 *μ*mol/L, respectively.

## 4. DISCUSSION

We have shown that this pannus model makes it much easier to determine a variety of cytokines produced in the granuloma than the previous methods which needs many steps to extract cytokines from tissue, and the type of cytokines is very similar to that reported to be increased in the pannus in the patients with RA. Antirheumatic drugs suppressed these cytokine productions and hydroxyproline contents. These results suggest that this model could be a relevant model for evaluation of pannus formation in patients with RA. Both PDE4 inhibitors, KF66490 and SB207499, showed an effect comparable to those of antirheumatic drugs in this model; and also, PDE4 inhibitors inhibited lipopolysaccharide-induced TNF-*α* and IL-12 production from thioglycolate-induced murine peritoneal macrophages and the proliferation of rat synovial fibroblasts. Therefore, PDE4 inhibitors could have therapeutic effects on pannus formation in RA by inhibition of cytokine production by macrophages and synovial fibroblast proliferation.

There was no report on the measurement of cytokines in delayed-type hypersensitivity granuloma. We compared cytokine concentrations between in-tissue homogenate samples and in-culture supernatants of the granuloma. However, a marked variability of cytokine concentrations was observed in the case of using homogenate samples (data not shown). The new method showed that the variation was relatively small and could be useful for the measurment of cytokine productions from granulomatous tissues.

A broad array of macrophages and fibroblast cytokines, including TNF-*α*, IL-1, IL-6, and IL-12 are produced by the rheumatoid synovium in the pannus [23–25]. TNF-*α*, IL-1*β*, and IL-6 have been reported to play a pivotal role in the pathogenic mechanisms of RA [26–28]. In this study, chronic production of TNF-*α*, IL-1*β*, IL-6, and IL-12 was observed. These observations suggest that this pannus model is useful for evaluating the effects of drugs on the production of cytokines involved in the pathogenic mechanisms of RA. PDE4 inhibitors suppressed these all cytokine production in vivo and TNF-*α* and IL-12 production in vitro. These results suggest that PDE4 inhibitors directly suppress the production of TNF-*α* and IL-12, and indirectly suppress the production of IL-1*β* and IL-6 which are possibly induced by TNF-*α* stimulation. Because it has been reported that TNF-*α* transgenic mice develop arthritis with enhanced production of TNF-*α*, IL-1, and IL-6 [29], which means TNF-*α* is the major dominant regulator of IL-1*β* and IL-6 [30]. Additional explanation of antipannus forming effects of PDE4 inhibitors is the inhibition of fibroblast proliferation. The maximum plasma concentration of SB207499 was about 19 *μ*mol/L at the maximum dose used in this study (30 mg/kg p.o.) [31]. Therefore, the inhibition of fibroblast proliferation is considered to be involved in antipannus forming effects of PDE4 inhibitors.

Some autoantibody levels in the synovial fluid are specifically related to the pathogenesis of RA [32, 33]. PDE4 inhibitors suppressed IgG_1_ but not IgM production in the pannus supernatants. This finding conflicts with previous reports that PDE4 inhibitors inhibit IgG_1_ or IgG_2a_ antibody production in vivo [34, 35], but they did not measure the antibody levels in the inflammatory sites. These results suggest that PDE4 inhibitors inhibit antibody production in the inflammatory sites. It is not known exactly why PDE4 inhibitors suppressed the IgG_1_ production; but in our model, IL-4 production on day 1 was completely inhibited by both PDE4 inhibitors (85 or 83% inhibition at 10 or 30 mg/kg of KF66490 and 53 or 103% inhibition at 10 or 30 mg/kg of SB207499). IL-4 is known to promote the proliferation and differentiation of activated B-cells. Therefore, the suppression of IL-4 production by PDE4 inhibitors may have had some inhibitory effects on B cells.

It has been reported that PDE4 inhibitors suppress both T_h_1 and T_h_2 cytokine production from stimulated T cells in vitro [36, 37]. However, the inhibition is partial. Therefore, we believe that the suppression of T cell function is less involved in antipannus effects of PDE4 inhibitors because no obvious inhibition of IFN-*γ* production was observed in our model (data not shown); and also, we did not find any effect on IFN-*γ* and IL-6 production from concanavalin. A stimulated murine splenic T cells up to 10 *μ*mol/L (data not shown). Interestingly, it has been reported that dendritic cells exposed to PDE4 inhibitors during maturation reduce the development of IFN-*γ*-expressing effector T cells [38]. Therefore, PDE4 inhibitors seem to affect the development of T_h_1 cells in vitro; but as mentioned above, IL-4 production was completely inhibited by PDE4 inhibitors in our model. The suppression of IL-4 production by PDE4 inhibitors might have had an impact on T_h_1/T_h_2 balance. Taken together, although PDE4 inhibitors preferentially diminish T_h_1 responses, some effects of PDE4 inhibitors on T_h_2 responses [36, 37] might complicate the in vivo responses. Consequently, we think the effects of PDE4 inhibitors on in vivo T_h_1/T_h_2 responses depend on the type of inflammation.

Recently, IL-17-producing T cells have been classified as a new effector T-cell subset, termed T_h_17, which is distinct from T_h_1, T_h_2, and Treg subsets. It is very interesting to examine whether PDE4 inhibitors suppresses the IL-23 or transforming growth factor (TGF-*β*) production from macrophages. IL-23, IL-6, and TGF-*β* promote the differentiation of T_h_17 cells, which are considered to act as the key effector-cell subset in inflammatory arthritis, at least in rodents [39].

In conclusion, this study provides a unique murine model to easily evaluate the effects of the drugs on the formation of the granuloma and cytokine production in a granuloma. PDE4 inhibitors showed an effect comparable to those of antirheumatic drugs in this model. The suppression of TNF-*α* and IL-12 production and the inhibition of fibroblast proliferation were considered to contribute to the antipannus forming effects of PDE4 inhibitors.

## Figures and Tables

**Table 1:** Summary of cytokine productions.

Drug	Dose (mg/kg)	TNF-*α*	IL-1*β*	IL-6	IL-12
		(% inhibition)			
KF66490	3	50**	33	−8	35**
	10	61**	60*	−41*	40**
	30	55**	80*	30	59***
SB207499	3	41*	39	14	25
	10	56**	65**	7	51***
	30	72***	86***	47*	72***
Leflunomide	3	67**	29	9	−4
	10	72**	48*	9	55*
	30	111***	71**	44**	43
Prednisolone	3	27	85*	59*	77*
	10	86***	60	65	87*
	30	107***	102**	93*	103**
Methotrexate	3	63	−68	41*	−17
	10	83**	37	53**	10
Diclofenac	1	−68*	−4	26	−37
	3	−77*	−9	51*	−92*

${}^{*}P<.05$, **P<.01, ***P<.001. Representative data from two or three independent experiments are shown.

**Table 2:** Summary of MPO activity, hydroxyproline content, anti-mBSA IgG_1_, and anti-mBSA IgM antibody production.

Drug	Dose (mg/kg)	MPO	Hydroxyproline	Anti-mBSA IgG_1_antibody	Anti-mBSA IgM antibody
		(% inhibition)			
KF66490	3	22	58*	34	9
	10	61*	37	39	33
	30	67*	75**	79*	34
SB207499	3	50*	61**	38	11
	10	51*	47*	81*	39
	30	83***	78***	61	34
Leflunomide	3	−20	46*	42	34
	10	36	62**	95*	86*
	30	80**	72***	104*	100*
Prednisolone	3	60	79**	68*	45
	10	9	81**	75*	55
	30	99*	100***	90*	67
Methotrexate	3	−22	9	47	40
	10	52	25	87**	91*
Diclofenac	1	−29	27	17	−86
	3	−66	−18	20	−120

${}^{*}P<.05$, **P<.01, ***P<.001. Representative data from two or three independent experiments are shown.

**Table 3:** IC_50_ values of KF66490 and SB207499 on LPS-induced cytokine production from thioglycolate-elicited peritoneal macrophages.

	TNF-*α*	IL-12
	(IC_50_: *μ*mol/L)	
KF66490	0.88	4.2
SB207499	0.12	0.52

Thioglycolate-induced adherent peritoneal cells were stimulated with 1 *μ*g/mL of LPS in the presence or absence of compounds for 6.5 h (for TNF-*α*) or 24 h (for IL-12) at 37^*°*^C. After the incubation, cytokines were measured by ELISA kits in the supernatants. Data are mean value of two or three independent experiments.

**Table 4:** IC_50_ values of various drugs on serum-induced proliferation of rat synovial fibroblasts.

Drug	IC_50_ (*μ*mol/L)
KF66490	9.2
SB207499	3.1
Leflunomide	*>*100
Prednisolone	0.070
Methotrexate	0.013

Rat synovial fibroblasts were pre-cultured in culture medium supplemented with 1% FBS for 24 h at 37^*°*^C. After the preculture, cells were stimulated by 10% FBS in the presence or absence of compounds for 48 h at 37^*°*^C followed by 24 h culture in the presence of 1.25 *μ*Ci/mL of [methyl-^3^H] thymidine or [6-^3^H] deoxyuridine. The incorporation of [^3^H] thymidine or [6-^3^H] deoxyuridine was counted by a liquid scintillation counter. Data are mean value of three or four separate determinations.
